# Patient experience of moderate asthma attacks: qualitative research in the USA and Germany

**DOI:** 10.1186/s41687-022-00506-2

**Published:** 2022-11-22

**Authors:** Maggie Tabberer, Jane R. Wells, Dale Chandler, Linda Abetz-Webb, Shiyuan Zhang, Wilhelmine Meeraus, Andy Fowler, David Slade

**Affiliations:** 1 Present Address: GSK, Brentford, Middlesex UK; 2 Patient-Centered Outcomes, Adelphi Values, Bollington, Cheshire UK; 3 Patient-Centred Outcomes Assessments Ltd, Bollington, Cheshire UK; 4 GSK Collegeville, 1250 S. Collegeville Rd, Collegeville, PA 19426 USA; 5 GSK, Research Triangle Park, NC USA

**Keywords:** Asthma, Moderate asthma exacerbation, Concept elicitation, Symptoms, Impact, Treatment, Health-related quality of life

## Abstract

**Background:**

There is limited information available on the impact of moderate asthma exacerbations, often called “asthma attacks” (i.e., those not requiring hospitalisation or treatment with systemic corticosteroids) on patients’ lives. This multi-country qualitative study explored the patient experience of these events.

**Methods:**

Semi-structured concept elicitation interviews were conducted in the USA and Germany with adult patients with asthma who had experienced a moderate asthma exacerbation in the prior 30 days. Physicians with experience in managing patients with asthma were also interviewed. Interviews explored patients’ experience of symptoms and impact of moderate exacerbations and associated exacerbation triggers and treatment patterns. Physicians were also asked about their interpretation of a clinical definition and treatment of a moderate exacerbation.

**Results:**

Twenty-eight patient (n = 20 in the USA, n = 8 in Germany) and six physician (n = 3 in the USA, n = 3 in Germany) interviews were conducted. During their moderate exacerbation, all patients reported experiencing shortness of breath, which many considered to be severe and the most bothersome symptom. Wheezing was also reported by all patients and considered severe by two thirds of patients. Most patients also reported coughing and chest tightness. All or almost all patients reported that moderate exacerbation caused fatigue/tiredness and impacted their physical functioning, emotional functioning, activities of daily living and work/school life. Most patients reported using rescue or maintenance inhalers to alleviate symptoms of the exacerbation. Conceptual saturation (i.e., the point at which no new concepts are likely to emerge with continued data collection) was achieved. Findings were used to develop a patient-focused conceptual model of the experience of moderate asthma exacerbations, outlining concepts related to triggers, symptoms, impact, and treatment from the patient perspective. Physician data was consistent with patient reports and complemented the conceptual model.

**Conclusions:**

Findings from concept elicitation interviews highlight the increased frequency, duration and severity of asthma symptoms and increased rescue medication use during moderate asthma exacerbations compared with the typical daily asthma experience, which have a substantial impact on patients’ lives.

**Supplementary Information:**

The online version contains supplementary material available at 10.1186/s41687-022-00506-2.

## Background

Asthma is a heterogeneous chronic inflammatory respiratory disease characterised by episodes of shortness of breath, wheezing, chest tightness and/or cough, and by variable expiratory airflow limitation that places a high burden on both patients and healthcare systems [1]. Patients with asthma may experience exacerbations of their symptoms and airflow limitation of varying severity, that impact health-related quality of life (HRQoL) and in some cases may be life-threatening [2–4]. Consequently, the Global Initiative for Asthma (GINA) and the USA National Institute of Health’s National Asthma Education and Prevention Program [5] have identified key long-term goals of asthma management, including good symptom control and minimizing the future risk of exacerbations, airflow limitation, hospitalisation, and side effects of treatment. To achieve these goals, a stepwise approach to asthma management is recommended, with treatment selection based on the severity of asthma and a continuous cycle of assessment, adjustment of treatment, and review of treatment response [1].

Although asthma exacerbations are typically more common and more severe when the underlying disease is uncontrolled or very poorly controlled [6–8], they can also occur in patients with otherwise mild or well-controlled asthma [1]. Exacerbation severity may be determined based on symptom changes, medication use, and/or other healthcare interventions. In 2009, a joint statement from the American Thoracic Society (ATS)/European Respiratory Society (ERS) on asthma control and exacerbations defined a moderate exacerbation as a deterioration in symptoms and lung function and increased rescue bronchodilator use lasting for ≥ 2 days [9]. According to the ATS/ERS statement, moderate exacerbations require a change in the treatment to prevent progression to a severe exacerbation, but they are not considered severe enough to warrant systemic corticosteroid use and/or hospitalization [9]. This definition also aligns with the European Medicines Agency guidance for clinical trials [10]. While the ATS/ERS definition is undoubtedly useful, characterization of the severity of an asthma exacerbation can be challenging in clinical practice because it is reliant in part on patient-reported deterioration in asthma control and changes in treatment that may not have been recorded in the clinic. This can be problematic because identification and appropriate treatment of a moderate asthma exacerbation may help to prevent its escalation to a severe event [11]. While steps have been taken to create a standard definition for the severity of asthma exacerbations, there remains substantial variability in the definitions used across research studies and a consensus on the most appropriate definition has not yet been reached.

A more specific, prospective definition of a moderate exacerbation is needed for use in clinical trials in order to more accurately measure the effect of a particular treatment on exacerbation risk [11]. Development of an accurate and consistent definition of a moderate asthma exacerbation requires in-depth information from the patients themselves on how these exacerbations manifest. There is limited available information regarding the impact of moderate exacerbations on patients and their daily lives. Furthermore, to the best of our knowledge, there is no published literature relating to the “lived experience” of moderate asthma exacerbations. To bridge this knowledge gap, qualitative interviews were conducted with adults with asthma and also with a group of physicians, to explore the patient experience of moderate asthma exacerbations. These findings will add to the understanding of the patient’s experience of the signs and symptoms of moderate exacerbations and the impact these have on functioning and HRQoL, as well as help to interpret existing measures of moderate exacerbations used in clinical studies.

## Methods

### Study design

This was a cross-sectional qualitative interview study conducted in the USA and Germany (GSK study ID: 209379). Participants included patients with moderate or severe asthma who had experienced a moderate asthma exacerbation within the past 30 days, and physicians with experience of managing patients with asthma. Participants took part in semi-structured concept elicitation telephone interviews for both patients and physicians and explored the patient experience of a moderate asthma exacerbation, including symptoms, exacerbation triggers, impact, and treatment. Ethical approval was obtained from Copernicus, a centralised Independent Review Board (IRB) in the USA with oversight for conduct of the study in the USA and Germany.

### Participant recruitment, screening, and eligibility criteria

#### Patients

Patients were identified by general practitioners, allergists, or pulmonologists from two regions in the USA (Chicago, IL and Los Angeles, CA) and one in Germany (Cologne and the surrounding North Rhine-Westphalia state). We originally intended the study to also include Spain; however, amendments were made to the protocol due to the ongoing COVID-19 pandemic, leading to cancellation of patient and clinician interviews in Spain. The interviews planned for Spain were reallocated to Germany and the USA. Patients were contacted by telephone or approached during scheduled or exacerbation-related appointments to determine if they had experienced a moderate exacerbation in the prior 30 days. Patients who wished to participate in the study provided written informed consent. The recruiting physician then completed the screener to provide additional details regarding the patients’ clinical information and medical history, which was then shared with a third-party recruitment agency who contacted the patient to collect demographic information and confirm eligibility. Recruitment quotas pertaining to age, sex, country, race, education, Asthma Control Test (ACT) score and medication step needed to maintain control were employed to ensure a diverse and representative sample of patients (Additional file 2: Table S1). All screened patients were therefore reviewed and approved prior to entering the study.

Eligible patients were aged ≥ 18 years with moderate or severe asthma at minimum maintenance treatment with inhaled corticosteroids (ICS)/long-acting β_2_-agonists (LABA) (GINA Step ≥ 2) and had experienced a moderate exacerbation within 30 days of recruitment. We aimed to recruit a total of 32 patients; if conceptual saturation was not achieved, we planned to conduct additional patient interviews. Patients were allowed to remain on their regular asthma maintenance medications. For recruitment purposes, a moderate exacerbation was defined as a deterioration in asthma symptoms, deterioration in lung function, or increased rescue bronchodilator use lasting ≥ 2 days that was not severe enough to warrant systemic corticosteroid use (or additional systemic corticosteroid use for those on regular systemic corticosteroid treatment) for > 2 days or an event that, when recognised, should have resulted in a temporary change in treatment in an effort to prevent the exacerbation from becoming severe.

To ensure the patients could recall their experience of the moderate exacerbation and not other conditions or events, patients were excluded if they had a history or current diagnosis of any clinically significant pulmonary diseases or abnormalities other than asthma (not including allergies or rhinitis), or had experienced a severe asthma exacerbation (defined as deterioration of asthma requiring the use of systemic corticosteroids for ≥ 3 days, and/or a hospitalisation/emergency department visit [not routine care]) within the past 90 days. Patients who were prescribed/had taken oral corticosteroids (OCS; or additional OCS for those on regular systemic corticosteroid treatment) to treat asthma for > 2 days within the past 30 days were excluded. Patients with a diagnosed onset of asthma at aged ≥ 40 years and current/former smokers (with a history of ≥ 10 pack years) who are more likely to have chronic obstructive pulmonary disease as a comorbidity were also excluded. Full eligibility criteria are provided in the Additional file 1: Supplementary Methods.

This study was conducted in 2020 during the COVID-19 pandemic. Patients with COVID-19 symptoms at screening would have been excluded as having “other respiratory conditions” and thus were ineligible to participate in the study. On the day of the interview, interviewers enquired if patients had developed COVID-19 symptoms during confirmatory screening before the interview. No patient interviews were rescheduled or terminated due to COVID-19.

#### Physicians

Third-party recruitment agencies identified and recruited physicians who then provided written informed consent. Eligible physicians specialised in managing and treating adult patients with asthma and regularly (i.e. monthly) saw patients with asthma. These physicians were not involved in recruiting patients for this study.

### Concept elicitation interviews

We used grounded theory methods to ensure that the resulting conceptual model best reflects patients’ experiences of moderate asthma exacerbations [12]. This approach also satisfied the guidance set out by the US Food and Drug Administration on best practice in conducting qualitative research and methods for eliciting information from patients [13]

The concept elicitation interviews lasted for 60 min and were conducted via telephone [14]. Interviews started with open-ended questions about a patient’s most recent exacerbation, providing opportunity for spontaneous report of concepts, followed by more focused questions to cover all concepts of interest. Patient interview topics included: language used to describe exacerbations, their duration, frequency and triggers, symptoms, impact on functioning and HRQoL, and steps taken to treat the exacerbation. Patients were asked about specific symptoms, shortness of breath, difficulty breathing, wheezing, coughing, chest tightness, phlegm/mucus, and chest pain if not discussed spontaneously. Comparison of the most recent exacerbation with more severe exacerbations (if experienced) was also discussed.

Physician interview topics included patients’ experiences of moderate asthma exacerbations, interpretation of a clinical definition of a moderate exacerbation, and patient treatment.

To ensure that all topics of interest were discussed, the interviews were conducted using a semi-structured guide, whereby development was led by experts in qualitative HRQoL research (Adelphi Values) with input from expert physicians. All interviews were audiorecorded and transcribed verbatim for the purpose of subsequent analysis. All identifiable information (e.g., names, locations) was removed from the transcripts such that they were fully pseudonymised. The German interviews were conducted by a native German speaker using a translated guide and also audiorecorded. The audiofiles were transcribed in German and then translated into English for subsequent analysis.

The findings from the patient interviews were used to develop a conceptual model outlining the patient experience and associated impact of moderate asthma exacerbations. The physician data was used to contextualise and support the patient results but was not used directly in the development of the model.

### Data analyses

Using grounded theory methods, interview transcripts were subject to thematic analysis through continuous data-driven coding and using Atlas.ti 8 software (Scientific Software Development GmbH, Berlin, Germany), a software package designed to facilitate the storage, coding, and analysis of qualitative data. Further information on thematic analysis and coding process is provided in the Additional file 1: Supplementary Methods. Patient sociodemographic and clinical characteristics were summarised using descriptive statistics (e.g., n values, means and min/max, range statistics). Conceptual saturation (i.e., the point at which no new concepts are likely to emerge with continued data collection) was monitored to confirm the adequacy of sample size [15, 16].

Analysis of the patient interview results was also conducted to explore these concepts in subgroups stratified by country (USA, Germany), sex (male, female), asthma severity (GINA Step 2, Step 3, Step 4), and allergies (yes, no).

## Results

### Patient characteristics

A total of 31 patients took part in the telephone interviews, 23 patients from the USA (between February and April 2020) and 8 patients from Germany (between September and October 2020). Three USA patients were excluded from the main analysis because descriptions of their asthma exacerbations during the interview violated the eligibility criteria (asthma exacerbations were too severe or too mild). All recruitment quotas were met (Additional file 2: Table S1).

Sociodemographic and clinical characteristics are shown in Table 1. The mean (standard deviation [SD]) age of the sample was 41.8 (15.8) years, there were slightly more females (n = 16; 57%), and the majority of patients were White/Caucasian/European (n = 20; 71%). The mean (SD) ACT score was 14.11 (3.49), indicating very poorly controlled asthma, and more than half the patients required GINA medication Step 4 or 5 to maintain asthma control. Allergies were the most common comorbidity. The most commonly prescribed maintenance medications were ICS/LABA single inhaler and leukotriene receptor antagonists.

**Table 1:** Patient sociodemographic and clinical characteristics

	USA (n = 20)	Germany (n = 8)	Total (n = 28)
Age, years, mean (SD)^a^	41.3 (16.3)	43.1 (15.6)	41.8 (15.8)
Sex, n (%)^a^			
Female	11 (55)	5 (63)	16 (57)
Race, n (%)^a^			
White/Caucasian/European	12 (60)	8 (100)	20 (71)
Mixed race	3 (15)	0	3 (11)
African American/African Heritage	2 (10)	0	2 (7)
Mexican	2 (10)	0	2 (7)
Native Hawaiian or other Pacific islander	1 (5)	0	1 (4)
Clinician-reported asthma severity level, n (%)			
Mild	2 (10)	0	2 (7)
Moderate	10 (50)	6 (75)	16 (57)
Severe	8 (40)	2 (25)	10 (36)
Age of asthma diagnosis, years, n (%)			
0–17	5 (25)	2 (25)	7 (25)
18–25	9 (45)	2 (25)	11 (39)
26–35	3 (15)	3 (38)	6 (21)
36– < 40	3 (15)	1 (13)	4 (14)
Asthma Control Test (ACT)^a^, at screening			
Mean (SD)	14.75 (3.6)	12.50 (2.9)	14.11 (3.5)
Well controlled (≥ 20), n (%)	5 (25)	0	5 (18)
Not well-controlled (16–19), n (%)	9 (45)	0	9 (32)
Very poorly controlled (≤ 15), n (%)	6 (30)	8 (100)	14 (50)
Medication step needed to maintain control, n (%)^a^			
GINA Step 2	8 (40)	0	8 (29)
GINA Step 3	3 (15)	2 (25)	5 (18)
GINA Step 4	9 (45)	5 (63)	14 (50)
GINA Step 5	0	1 (13)	1 (4)
Comorbidities, n (%)			
Allergies	5 (25)	7 (88)	12 (43)
Allergic rhinitis	5 (25)	3 (38)	8 (39)
Cardiac disorders	2 (10)	0	2 (7)
Blood and lymphatic system disorders	1 (5)	0	1 (4)
Osteoarthritis—‘back issues’	1 (5)	0	1 (4)
Metabolism and nutrition disorders	1 (5)	0	1 (4)
Respiratory, thoracic, and mediastinal disorders	0	1 (13)	1 (4)
Skin/subcutaneous tissue disorders	0	1 (13)	1 (4)
Psychiatric disorders	0	1 (13)	1 (4)
Maintenance medication, n (%)			
Leukotriene receptor antagonist	8 (40)	4 (50)	12 (43)
ICS/LABA (single inhaler)	12 (60)	5 (63)	17 (61)
ICS/LABA/LAMA (single inhaler)	0	3 (38)	3 (11)
ICS	7 (35)	0	7 (25)
LABA	0	1 (13)	1 (4)
Monoclonal antibody	3 (15)	6 (75)	9 (32)
LAMA/LABA	2 (10)	0	2 (7)
LAMA	0	3 (38)	3 (11)
SABA	1 (5)	1 (13)	2 (7)

^a^Variables used for patient sampling quotas. GINA, Global Initiative for Asthma; ICS, inhaled corticosteroid; LABA, long-acting β_2_-agonist; LAMA, long-acting muscarinic receptor antagonist; SABA, short-acting β_2_-agonist; SD, standard deviation

### Description, duration, and frequency of moderate exacerbations

Just under half of patients described their recent exacerbation as an “asthma attack” with fewer patients using the term “flare up” (Table 2). No patients spontaneously referred to their experience as an exacerbation. Patients most frequently reported that the exacerbation lasted 2–3 days (Table 2). A substantial number of patients reported that they had experienced moderate exacerbations once every 1–2 months or once every 3–4 months (Table 2), as a patient highlighted when asked, “*How frequently do you get these flare-ups?” “Um, not, not regularly. I’d say maybe every couple months.*” (US-05).

**Table 2:** Patient description of moderate asthma exacerbations

	USA (n = 20)	Germany (n = 8)	Total (n = 28)
Terms/adjectives used^a^, n (%)			
Asthma attack	12 (60)	0	12 (43)
Flare up	3 (15)	5 (63)	8 (29)
Deterioration	0	2 (25)	2 (7)
Episode	1 (5)	0	1 (4)
Black day	1 (5)	0	1 (4)
Difficulty breathing	0	1 (13)	1 (4)
Pushed too hard	1 (5)	0	1 (4)
Scary	3 (15)	0	3 (11)
Aggravating	1 (5)	0	1 (4)
Duration (start to recovery), n (%)			
2 days	6 (30)	1 (13)	7 (25)
2–3 days	4 (20)	1 (13)	5 (18)
3 days	3 (15)	2 (25)	5 (18)
3–4 days	2 (10)	0	2 (7)
6 days	0	1 (13)	1 (3)
5–10 days	2 (10)	3 (38)	5 (18)
1 week	1 (5)	0	1 (3)
1 month	1 (5)	0	1 (3)
Unclear	1 (5)	0	1 (3)
Frequency, n (%)			
Every 3 days	1 (5)	0	1 (3)
2–4 times a month	4 (20)	1 (13)	5 (18)
Once every 1–2 months	5 (25)	2 (25)	7 (25)
Once every 3–4 months	5 (25)	0	5 (18)
Once or twice a year	2 (10)	2 (25)	4 (14)
Once every 2 years	1 (5)	0	1 (3)
Varies during the month	1 (5)	0	1 (3)
Varies depending on the season	1 (5)	1 (13)	2 (7)
Not reported	0	2 (26)	2 (7)

^a^Two patients reported more than one descriptor

Patients reported being in recovery from an asthma exacerbation when having residual/persisting symptoms that were worse than typical, but no longer acutely part of the exacerbation. One patient replied that the moderate exacerbation lasted”*probably three to four days”* when asked “*So, how long did this last if you had to gauge from right from the start until you felt completely back to normal, how long did this asthma attack last for you?”* (US-03).

The reported durations consider the patients’ perceived full length of the exacerbation from onset to full recovery.

### Triggers of moderate asthma exacerbations

Patients reported one or more triggers of their exacerbation. The most frequently reported triggers for a moderate exacerbation were environmental factors, such as change in weather, cold air, and humidity; followed by exercise or other kinds of physical activity; allergens such as pets; emotions; and irritants, such as poor air quality. Few patients reported respiratory infection/common cold as triggers. A patient described the trigger of an exacerbation: “*I actually think that was this change in the weather again. So, from the warm season back to the somewhat cooler [weather] again. Because I always have problems anyway with dry air due to the central heating*.” (Germany-29).

### Symptoms of moderate asthma exacerbations

**Fig. 1:**
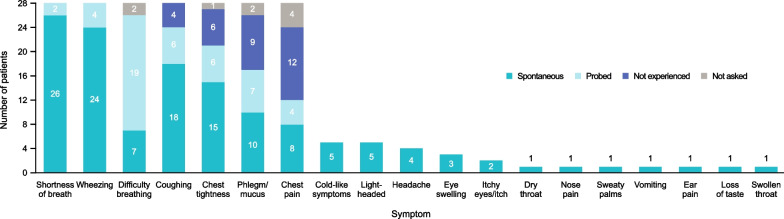
Symptoms of moderate asthma exacerbations.* Note*: Shortness of breath, wheezing, difficulty breathing, coughing, chest tightness, phlegm/mucus, and chest pain were probed by the interviewer. All other symptoms were only reported spontaneously and because of that, corresponding columns are nonstacked

An overview of reported moderate exacerbation symptoms is shown in Fig. 1; patient quotes describing why symptoms were bothersome are shown in Table 3. Shortness of breath was reported by all patients as a symptom of the exacerbation and by almost all patients spontaneously. Difficulty breathing was considered conceptually the same as “shortness of breath” by the majority of patients and by all the physicians. Patients considered shortness of breath to be severe or moderate to severe during the exacerbation. Duration ranged from a few minutes until medication was taken, to the full 3 days of the exacerbation to recovery with most patients reporting shortness of breath lasting for up to 3 h during the exacerbation. Shortness of breath was reported to be the first symptom experienced by the majority of patients or to occur within the first 30 min of the onset of the exacerbation and prompted many patients to use rescue/additional maintenance inhaler. Shortness of breath was also reported to be the most bothersome symptom in the majority of patients, as described by one patient: *“But when this shortness of breath comes, especially if it’s the middle of the day, then it’s super disruptive.”* (Germany-31).

**Table 3:** Patient-reported most bothersome symptoms^a^ during the exacerbation

Symptom reported as bothersome^b^	Reason (where given) for why symptom reported as bothersome	Supporting quotes
Shortness of breath/difficulty breathing (n = 21)	Worry/anxiety (n = 3)	*“Um, it just causes me like the most worry and um, yeah, I guess that’s, that’s why that makes it the most bothersome.”* (USA-03)
	Fear (n = 3)	*“But when this shortness of breath comes, especially if it’s the middle of the day, then it’s super disruptive.”* (Germany-31)
	Limits activity (n = 3)	*“****And why was that the most bothersome?**** Just because it’s really hard to breathe.”* (USA-03)
	Panic (n = 2)	
	Hard to breathe (n = 2)	
	Disruptive (n = 1)	
	Annoying (n = 1)	
	Like suffocating (n = 1)	
	Scary (n = 1)	
Coughing (n = 6)	Aggravating (n = 1)	*“…probably the coughing cuz it’s really, it’s like really an intense cough.”* (USA-02)
	Intense (n = 1)	*“It just gets a harder and harder cough where it makes my chest feel very sore at the end.”* (USA-04)
	Hard coughing leads to chest pain (n = 1)	*“****What was the most disruptive thing?**** The coughing.”* (Germany-30)
	Self-conscious (n = 1)	
	Disruptive (n = 1)	
Chest tightness (n = 6)	Cannot continue with daily tasks (n = 1)	*“That what makes me feel that sometimes I cannot continue on my daily… it bothers me when I’m doing my daily tasks.”* (USA-08)
	Disruptive (n = 1)	*“The feeling of tightness in my chest”* (Germany-26)
Wheezing (n = 4)	Self-conscious (n = 1)	*“Everybody can hear you wheezing.”* (USA-01)
	More persistent than other symptoms (n = 1)	*“****Why the wheezing?**** It was more frequent.”* (USA-11)
	Limits activities (n = 1)	
Chest pain/discomfort (n = 2)	Takes a lot out of the patient (n = 1)	*“So, like the chest pain, um, that’s just… That, that takes a lot out… So that’s usually the one that I want, want to go away the most.”* (USA-13)

^a^A symptom was considered most bothersome based on the concept's intensity, frequency, disruption of daily life and cause for worry/panic; ^b^n corresponds to the number of patients who considered the symptom most bothersome, where given

Wheezing was also reported by all patients, the majority of whom reported the symptom spontaneously. Wheezing was reported to be one of the first symptom to occur by few patients and usually in conjunction with shortness of breath. Patients considered the symptom to be severe or moderate during the exacerbation, ranging from a few minutes to the length of the moderate exacerbation.

The majority of patients reported shortness of breath and wheezing as part of their typical day with asthma, but when part of an exacerbation, patients reported the symptom as more frequent, lasting longer, and more severe as indicated by a patient: “…it was like a very drowning, a very drowning sensation, like, like I said, um, you just can’t breathe and it just, um, overpowers everything that you have going, I’m trying to, you know, gasp for breath and it’s hard.” (US-04).

Most patients also reported coughing (with many patients reporting this spontaneously), chest tightness (with just over half of patients reporting this spontaneously) and phlegm/mucus (with less than half of patients reporting this spontaneously). The symptoms of a moderate exacerbation was described as: *“It just gets a harder and harder cough where it makes my chest feel very sore at the end.”* (US-04).

### Impact of moderate asthma exacerbations

**Fig. 2:**
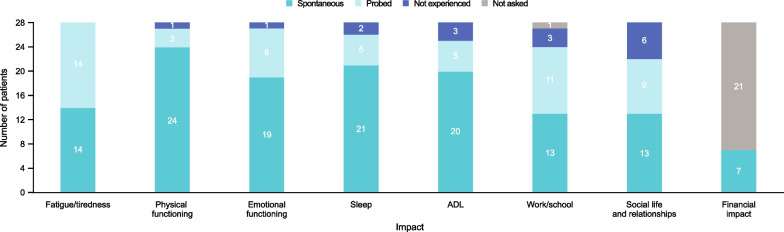
Impact of moderate asthma exacerbations.* ADL* activities of daily living

All patients reported that their moderate exacerbation resulted in fatigue/tiredness, with half of patients reporting this spontaneously), and almost all patients reported impact on physical functioning (exercising, walking, and climbing stairs) (n = 27; n = 24 spontaneously) and emotional functioning (n = 27; n = 19 spontaneously). Anxiety/worry was reported by the majority of patients and anxiety/stress was associated with an exacerbation of symptoms in over a third of patients. The majority of patients reported impact on sleep (n = 26; n = 21 spontaneously), which included nighttime awakenings and difficulty falling asleep. Shortness of breath and coughing were most frequently reported as causing the biggest impact on sleep. Most patients also reported impact on activities of daily living (ADL) (n = 25; n = 20 spontaneously); social life and relationships (n = 22; n = 13 spontaneously) including ability to participate in social activities; and impact on work/school, such as absences. Fewer patients reported a financial impact due to their recent exacerbation (none spontaneously). Additionally, some patients identified coughing as a symptom that was bothersome and had social and work impact. Although not identified as severe or as bothersome as shortness of breath, coughing caused an irritation to patients during the course of the moderate exacerbation. However, some patients also reported that coughing was related to phlegm/mucus and chest pain/discomfort, with chest pain/discomfort being reported as a bothersome symptom for the few patients who did experience it. Figure 2 summarises the number of patients reporting each impact spontaneously and when probed by the interviewer; patient quotes describing the impact of exacerbations are shown in Table 4. The most impactful aspects of a moderate exacerbation were primarily related to symptoms, with shortness of breath/difficulty breathing the most frequently reported symptom as described by a patient who said: *“Yes, the difficulty breathing, in other words, the shortness of breath, that is the worst thing of all.”* (Germany-26).

**Table 4:** Patient quotes of the most impactful aspect of a moderate asthma exacerbation

Findings	Example quote
Patients described the most impactful aspect of a moderate asthma exacerbation **Symptoms:** Shortness of breath/difficulty breathing (n = 12) Wheezing (n = 2) Coughing (n = 2) Chest tightness (n = 2) Chest pain (n = 1) **Impact:** Sleep disturbance (n = 3) Anxiety/worry about an attack (n = 2) Physical impact (general) (n = 2) Stops your day (n = 1) Fatigue/tiredness (n = 1)	*“****Which aspect of the asthma attack would you say is the most impactful for you?**** I think it’s the shortness of breath overall.”* (USA-10) *“Just being fearful of having an attack in public or somewhere where I’m not around anyone who’s going to help me, or in the car…just being always fearful of when you can have an attack”* (USA-09) *“Yes, the difficulty breathing, in other words, the shortness of breath, that is the worst thing of all.”* (Germany-26) *“It has to be the chest tightening, the chest tightening with the short of breath. That always impacts me more.”* (USA-08) *“it’s still kind of like a, like an inconvenience, um, to not breathe and the anxiety that comes with not breathing…but usually it’s the chest pain. The chest pain is the one that, like, I… that’s not fun at all.”* (USA-13)

### Comparison of recent exacerbation to a previous worse exacerbation

When asked to compare the worst exacerbation they have ever had to their recent exacerbation, the majority of patients reported having experienced a worse exacerbation, and almost half of patients reported that the worse exacerbation lasted longer, ranging from 3 days to 2 months. Patients reported that the symptoms of the worse exacerbation were more severe including shortness of breath, chest pain/tightness, wheezing, or that symptoms in general were more severe. Patients most commonly reported that different from their most recent moderate exacerbation, they were prescribed a course of OCS during the worse exacerbation and/or that they were hospitalised.

### Treatment steps for moderate asthma exacerbations

**Fig. 3:**
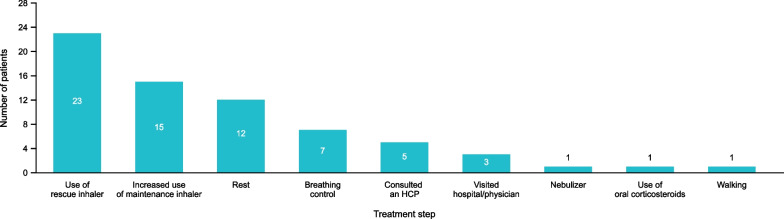
Pharmacological and nonpharmacological management reported by patients for their moderate asthma exacerbations.* HCP* healthcare professional

Most patients reported using a rescue inhaler (short-acting β_2_-agonist), with more than half of patients increasing the use of a maintenance inhaler to alleviate their symptoms and the impact of a moderate exacerbation. Some patients consulted an healthcare professional (HCP) or visited a hospital or a physician. Some patients also reported nonpharmacological management, including rest, breathing control, and walking (Fig. 3). Approximately a third of patients reported having an asthma action plan in place with their HCP to manage an asthma exacerbation.

### Subgroup analyses

#### Exacerbation symptoms stratified by country, sex, asthma severity, and presence of allergies

Exacerbation symptoms reported were consistent across the country, sex, asthma severity, and allergy subgroups explored. Minor differences (e.g., feeling light-headed was only reported in the USA and eye swelling was only reported in Germany) in some reported symptoms were observed between countries though all core symptoms were consistent, and the impact of moderate exacerbations were similar across both the USA and German patient samples.

### Conceptual saturation

Conceptual saturation for patient interviews was achieved with the 31 patients recruited: most concepts were spontaneously elicited before the last set of five interviews (36/37 concepts; 97%), with 76% of concepts (28/37) identified in the first three sets of interviews and 95% of concepts (35/37) identified by the fourth set (Additional file 1: Fig. S1A). For the physician interviews, the majority of the concepts were spontaneously elicited before the last set of two interviews (15/16 concepts; 94%) (Additional file 1: Fig. S1B). These results show that new concepts were unlikely to emerge with further interviews and indicates adequacy of the sample size.

### Conceptual model

**Fig. 4:**
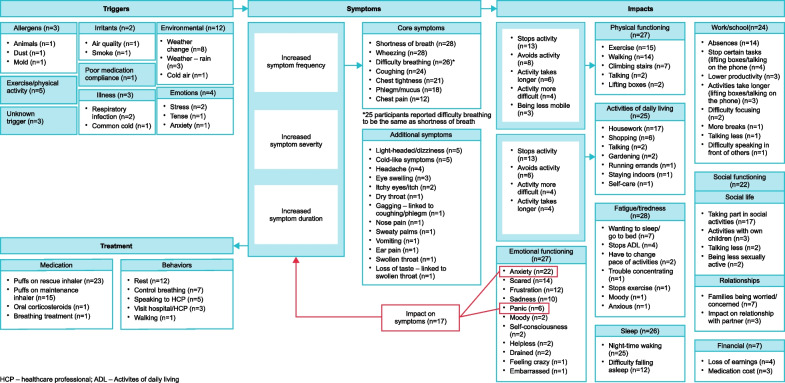
Conceptual model of the patient experience of a moderate asthma attack (moderate exacerbation)^a^. ^a^Model was developed based on interviews with 8 German and 20 USA patients and on ATS/ERS definition of a moderate exacerbation. *ATS* American Thoracic Society;* ERS* European Respiratory Society

Findings from the interviews were used to develop a patient-focused conceptual model showing patient experience of the triggers, symptoms, treatment, and impact of moderate exacerbation and the links between these (Fig. 4).

### Physician interviews

Six physicians participated in the qualitative interviews, four pulmonary specialists (one from the USA and three from Germany) and two allergists (both from the USA). Physicians working in community and public hospitals (n = 2), private practices (n = 3), and public/private practices (n = 1) were represented. Physicians had a mean (SD) of 19 (8.6) years of experience managing patients with asthma.

Similar to the patients’ reports, four physicians noted that their patients most commonly described their moderate exacerbations in terms of specific symptoms (e.g., shortness of breath, chest tightness, and difficulty breathing). Two reported the use of the term “asthma attack” and one the use of “flare” and, four physicians reported that their patients would not use the term “exacerbation.” Physicians tended to report that moderate exacerbations were lengthier in duration compared with patients (2–3 days), with three physicians reporting that a moderate exacerbation would last 3–5 days on average and three adding that the moderate exacerbation could last up to 2 weeks.

In addition, physicians reported that moderate exacerbations occurred less frequently than reported by patients, with half of the physicians reporting that they occurred about 2–3 times per year (as opposed to 3–12 times per year as reported by patients). Allergens were reported to be a major trigger for moderate exacerbations by all physicians followed by illness (cold and flu) reported by four physicians.

Symptoms and impact of moderate exacerbations reported by physicians were generally the same as those reported by patients; shortness of breath, difficulty breathing, coughing, chest tightness, and phlegm/mucus were reported by all physicians, as were impact on sleep, physical functioning, work/school, ADL, social life and relationships, emotional functioning, fatigue/tiredness, and finances. Similar to findings from patient interviews, all the physicians reported that shortness of breath and difficulty breathing were the same concept. Treatment steps physicians would expect a patient to take to treat their moderate exacerbation were also in accordance with patient findings, with rescue inhaler and maintenance inhaler both mentioned by four physicians. However, in contrast to patient findings (9 patients; 32%), all physicians reported that their patients would have an asthma action plan in place developed with their physician to manage their asthma exacerbation.

## Discussion

This qualitative study characterised the patient experience of moderate asthma exacerbations and the resulting conceptual model illustrates patient experience of exacerbations, including the triggers, symptoms, impact, and treatments and connections between these concepts. Findings from the physician interviews were generally consistent with patient reports. The conceptual model illustrates that patients experience both a deterioration in symptoms and increased rescue bronchodilator use during a moderate exacerbation, which is consistent with the joint ATS/ERS definition of a moderate exacerbation [9]. Further, it provides an in-depth characterization of symptoms and their debilitating impact on patients’ HRQoL.

Our aim of including both North American and European participants was to ensure we obtained a breadth of patient and physician insight into moderate exacerbations and their impact, which may vary according to location, and be influenced by language and cultural factors. Conducting this research in a North American country and in Europe enabled the research to explore similarities and differences in the patient experience between these two countries***.***

During the interviews, patients reported a core set of symptoms that included shortness of breath (difficulty breathing) and wheezing, experienced by every patient, as well as one or a combination of coughing, chest tightness, phlegm/mucus, and chest pain/discomfort. In line with the ATS/ERS definition of a moderate exacerbation [9], patients in the current study reported that the symptoms of shortness of breath and wheezing, in particular, differed from their typical experience of these symptoms during the moderate exacerbation event, specifically presenting with a higher level of severity, greater frequency, and longer duration. Building on the identification of symptoms associated with a moderate exacerbation, patients often reported the point at which their exacerbation symptoms were experienced in comparison with other symptoms, suggesting that the order in which symptoms manifest was important to them. Shortness of breath (difficulty breathing) and wheezing were most frequently reported as the first symptoms experienced during the moderate exacerbation. Interestingly, while most patients reported shortness of breath to be the most bothersome symptom during the moderate exacerbation, wheezing was not considered as bothersome despite being equally prevalent among the patients. This may be because shortness of breath is more frightening to patients when compared to wheezing. This is consistent with a qualitative interview study conducted in patients with asthma where shortness of breath was the most difficult symptom reported by patients followed by chest tightness, coughing, and wheezing [17]. The order of exacerbation symptoms noted here may be helpful for patients and physicians to discriminate moderate exacerbations from asthma daily symptoms, and also to decide treatment needs during a moderate exacerbation.

In this study, the majority of patients and all physicians reported that shortness of breath and difficulty breathing were conceptually the same and, based on this, these two concepts were described together and interpreted as synonymous. In contrast, previous patient-centered research that explored this in-depth with adults and adolescents with asthma described “shortness of breath” and “difficulty breathing” as being related and suggested they can be grouped together with wheezing under “breathing symptoms” but are conceptually distinct [18].

While in-day and day-to-day variability of exacerbation symptoms were explored with probes during the interviews, these concepts were not spontaneously reported to any great extent by patients. Because the interviews were based on patient recall of symptoms, real-time data capture using an app may be a useful tool to explore this concept in the future.

A key objective of this study was to explore the impact of moderate exacerbation symptoms on patients’ HRQoL. Shortness of breath, as the most common exacerbation-related symptom, was often cited as having the greatest impact. Shortness of breath limited patients’ physical functioning and was also a common cause of emotional impact, often eliciting a sense of anxiety and fear, which in turn was often reported to further exacerbate the symptoms such as shortness of breath. The range of impact reported as a result of a moderate exacerbation in this study reflects daily patient-reported experiences of asthma in the literature [19, 20] highlighting that, as with symptoms, day-to-day impact are exacerbated during a moderate asthma exacerbation.

Patient–physician discordance regarding the frequency and severity of asthma symptoms has been reported in the literature [21, 22]. In the current study, physicians tended to report lengthier but less frequent moderate exacerbations than did the patients. Physician data were consistent with the mean duration of 11.1–12.1 days reported for a moderate exacerbation in the CAPTAIN study, which assessed the annualised rate of moderate and/or severe asthma exacerbations (key secondary endpoint) [23]. These differences between patients and physicians’ perception on the length of a moderate exacerbation could perhaps be attributed to patients defining the end of an exacerbation as the time when they are able to resume activities as opposed to when their symptoms return to pre-exacerbation levels. Additionally, there was a discordance in frequency with patients experiencing three times as many moderate exacerbations than reported by physicians. This discrepancy could perhaps be associated with the fact that patients may not often seek care for what they perceive as a moderate exacerbation, and as a result could be self-treating. These results suggest that moderate asthma exacerbations are underrecognised in clinical practice.

Patient interviews highlighted that the most common terms used by patients to describe an asthma exacerbation were “asthma attack” and “flare up.” This suggests that while the exacerbation did not meet the definition of severe in terms of required treatment, patients regarded this event as distinct from daily experience of asthma and also disruptive of their daily life. Although, none of the patients used the term “exacerbation” to describe the event, the concept of moderate exacerbation was consistent with the patients’ experience of an “asthma attack” or “flare up.” This suggests that it may be beneficial for clinicians to consider the terminology used to describe a moderate exacerbation with their patients.

Only a third of patients reported that they had an asthma action plan in place with their HCP, highlighting a potential need to seek additional treatment for an exacerbation in these patients. While all of the physicians interviewed reported that their patients would have such a plan, this is likely to be related to their specialist practice. As asthma action plans are generally used to cover symptom variability, and moderate exacerbation treatment is subsumed under general asthma management, there might be a need for a treatment specific plan to handle moderate exacerbations. Despite the reported differences between patient and physician perceptions of the patient experience of a moderate exacerbation, in general the symptoms and impact reported by patients were also reported by physicians, supporting their clinical relevance and the relevance of the ATS/ERS definition of a moderate exacerbation [9]. Notably, this definition was applied in recent Phase III clinical trials (including CAPTAIN) that were part of the network meta-analysis studying the benefits of triple therapy in patients with uncontrolled asthma. The CAPTAIN study assessed the effects of once-daily single-inhaler triple therapy with an ICS/LABA/long-acting muscarinic receptor antagonist (fluticasone furoate/umeclidinium/vilanterol) in patients with inadequately controlled asthma despite ICS/LABA treatment [23]. In our study and in CAPTAIN, the ATS/ERS definition was adapted slightly to include an increase in systemic corticosteroids (less than double the maintenance dose) for patients who were already receiving systemic corticosteroid treatment. While systemic corticosteroid use is usually associated with severe exacerbations [9], this change was made to reflect the use of systemic corticosteroids in clinical practice for exacerbations that would otherwise be defined as moderate. In this study, only one patient reported taking OCS (for ≤ 2 days) to treat their moderate exacerbation. In the CAPTAIN study, changes in lung function were similar for moderate and severe exacerbations while symptom scores were slightly higher for severe exacerbations [24].

The study had a number of strengths including a sample with demographic and clinical diversity in terms of age, sex, race, education level, disease severity and control, and treatment step. As conceptual saturation is typically achieved in as few as 12 individual interviews in a relatively homogenous population [15, 16], a sample of 28 was adequate to fulfil study objectives. Indeed, in our study, almost all spontaneously reported concepts were mentioned before the final set of interviews. As such it is unlikely that any additional interviews in a similar population would reveal further concepts. Nonetheless, there are some study limitations to consider. First, this was a qualitative study and the extent to which any qualitative research can be generalised should be considered. The findings obtained from the participant interviews cannot necessarily be extrapolated or generalised to the wider asthma population. The asthma population included those with moderate/severe asthma who were treated with ICS/LABA (with or without additional controller therapies) and those with diagnosed asthma onset younger than age 40 years. While this ensured that only moderate exacerbations due to asthma were captured, results from this specific population are not necessarily generalizable to the wider asthma population. Furthermore, smokers were excluded from participation in this study to also minimise smoking as a potential confounding factor. It should, however, be recognised that it is reported that around one-fifth of patients with asthma do smoke and that differences in the way patients experience moderate asthma exacerbation may exist between smokers and nonsmokers. The timing of the study is also an important factor because interviews were conducted during the global COVID-19 pandemic. This is likely to have changed patients’ usual daily activities and may have affected their experience with exacerbations given that some may have had increased anxiety during this period and access to their usual healthcare resources may have been impacted, although this was not reported in interviews. Finally, although our study gives significant insights into how patients are impacted by moderate exacerbations, it only included patients from two countries. Patient interviews in other countries and regions (e.g., Asia) would be useful in the future to ensure cross-cultural applicability of our results, and to study the impact of seasonality on moderate asthma exacerbations in other geographic areas because patients frequently reported environment and allergens as triggers of these events.

## Conclusions

Findings from these qualitative interviews highlight that the frequency, severity, and duration of core asthma symptoms increase during a moderate asthma exacerbation, as does rescue/maintenance medication use. Moderate exacerbations result in fatigue/tiredness and impact sleep, physical and emotional functioning, and ADL, and that can lead to emotional distress. Given their impact on patients and cost to healthcare systems, moderate exacerbations should be given greater prominence. With a minor adaptation (increase in the maintenance dose of OCS), the conceptual model is consistent with the definition of a moderate exacerbation in the ATS/ERS guidelines [9], and provides an in-depth patient perspective on the symptom and HRQoL impact experienced during a moderate asthma exacerbation.

## Supplementary Information

**Additional file 1**. Supplementary Methods.

**Additional file 2**. Table S1.

## Data Availability

Pseudonymized individual participant data and study documents can be requested for further research from http://www.clinicalstudydatarequest.com.

## Abbreviations

ACT: Asthma Control Test
ADL: Activities of daily living
ATS: American Thoracic Society
ERS: European Respiratory Society
GINA: Global Initiative for Asthma
HCP: Healthcare professional
HRQoL: Health-related quality of life
ICS: Inhaled corticosteroids
IRB: Independent Review Board
LABA: Long-acting β_2_-agonists
OCS: Oral corticosteroids
SABA: Short-acting β_2_-agonist
SD: Standard deviation
