# Supplementary material for: Patient experience of moderate asthma attacks: qualitative research in the USA and Germany
Source: J Patient Rep Outcomes. 2022 Nov 22;6:117. doi: 10.1186/s41687-022-00506-2 (PMC9681943; doi:10.1186/s41687-022-00506-2)
Supplement: Supplementary file 1 — Additional file 1. Supplementary Methods. [file 41687_2022_506_MOESM1_ESM.docx]

**SUPPLEMENTARY MATERIALS**

**Supplementary Methods**

#### Patient inclusion criteria

Patients were required to meet the following inclusion criteria to be eligible for inclusion in the study.

1. Male or female aged ≥18 years.
2. Diagnosis of moderate or severe asthma and is consistently treated with inhaled corticosteroids (OCS) / long-acting β2-agonists, as defined by national or international guidelines (e.g., National Institutes of Health 2007 Expert Panel Report 3 [EPR 3] or Global Initiative for Asthma [GINA])[5,1].
3. Experienced a moderate exacerbation within 30 days of recruitment, defined as:

- A deterioration in asthma symptoms, deterioration in lung function, or increased rescue bronchodilator use lasting for at least 2 days or more but that was not severe enough to warrant systemic (tablets, suspension, or injection) corticosteroid use (or additional systemic corticosteroid use for those on regular systemic corticosteroid treatment) for more than 2 days.
- An event that, when recognised, should have resulted in a temporary change in treatment, in an effort to prevent the exacerbation from becoming severe.

1. Personally signed and dated a written informed consent form prior to admission to the study.
2. Verbally fluent and literate in the language of the country of recruitment.
3. Patient was, to the best of the recruiting clinician’s knowledge, physically and cognitively capable to participate in a 60-minute telephone interview about their experience with a moderate asthma exacerbation.
4. Based on the EPR 3 guidelines (or equivalent, e.g., GINA )[1,5], the lowest level of treatment required to maintain the patient’s control of asthma was at least Step 2.

#### Patient exclusion criteria

Any patient presenting with any of the following at screening were not included in the study.

1. History, clinical suspicion, or current diagnosis of any clinically significant pulmonary diseases or abnormalities other than asthma (not including allergies or rhinitis), including:
   - Chronic obstructive pulmonary disease
   - Pneumonia
   - Active tuberculosis
   - Lung cancer
   - Significant bronchiectasis
   - Sarcoidosis
   - Lung fibrosis
   - Pulmonary hypertension
   - Interstitial lung diseases
   - Or other disease(s) that (in the investigator’s opinion) is likely to affect the patient’s experience of asthma.
2. Had a severe asthma exacerbation within the past 90 days, defined as deterioration of asthma requiring the use of systemic corticosteroids (tablets, suspension, or injection) for 3 days or more and/or required a hospitalization/emergency room visit (not routine care). This exclusion was to avoid conflation in descriptions of the effect of the recent moderate exacerbation with the experience of a severe exacerbation.
3. Prescribed/took OCS (or additional OCS for those on regular corticosteroid treatment) to treat asthma for more than 2 days, within the past 30 days prior to recruitment.
4. Diagnosed onset of asthma at aged ≥40 years.
5. Current smoker (defined as a patient who has used inhaled tobacco products within the 12 months prior to recruitment [i.e., cigarettes, e-cigarettes/vaping, cigars, or pipe tobacco]), or a former smoker with a smoking history of ≥10 pack years (e.g., ≥20 cigarettes/day for 10 years).
6. Currently enrolled, or had been enrolled, within the previous 30 days of recruitment, in a clinical trial.

**Data analyses**

Using a grounded theory approach as the foundation, the thematic analysis was an iterative process which started with coding the open-ended questions that were used to elicit concepts without probing for specific symptoms and/or experiences by the interviewers, followed by those concepts probed by the interviewers. Codes were created as new concepts emerged.

The ATLAS.ti software package (Scientific Software Development GmbH, Berlin, Germany) allows traditional qualitative analysis of transcripts and facilitates easier breakdown and management of qualitative data into groups and assignment codes. The first two transcripts from different countries were coded by two members of the coding team separately, followed by a consensus on a provisional code list and coding scheme. Patient and clinician transcripts were coded separately, with a separate code list generated for each. Thereafter, each transcript was coded by individual members of the research team, with selected transcripts checked by the project leader. Prior to coding an interview, each coder read through the transcript in its entirety to gain a clearer picture of the participants’ context and experience. Any important points were highlighted in memos in ATLAS.ti. After analysing each transcript, the coded statements were moved into their relative domains. A list of statements was generated for each domain, all of which contained a prefix comprising the participant’s designated ID number

**Supplementary Figures**

**Supplementary Fig. 1:** **Conceptual saturation from the patient (A) and physician (B) interviews**

**
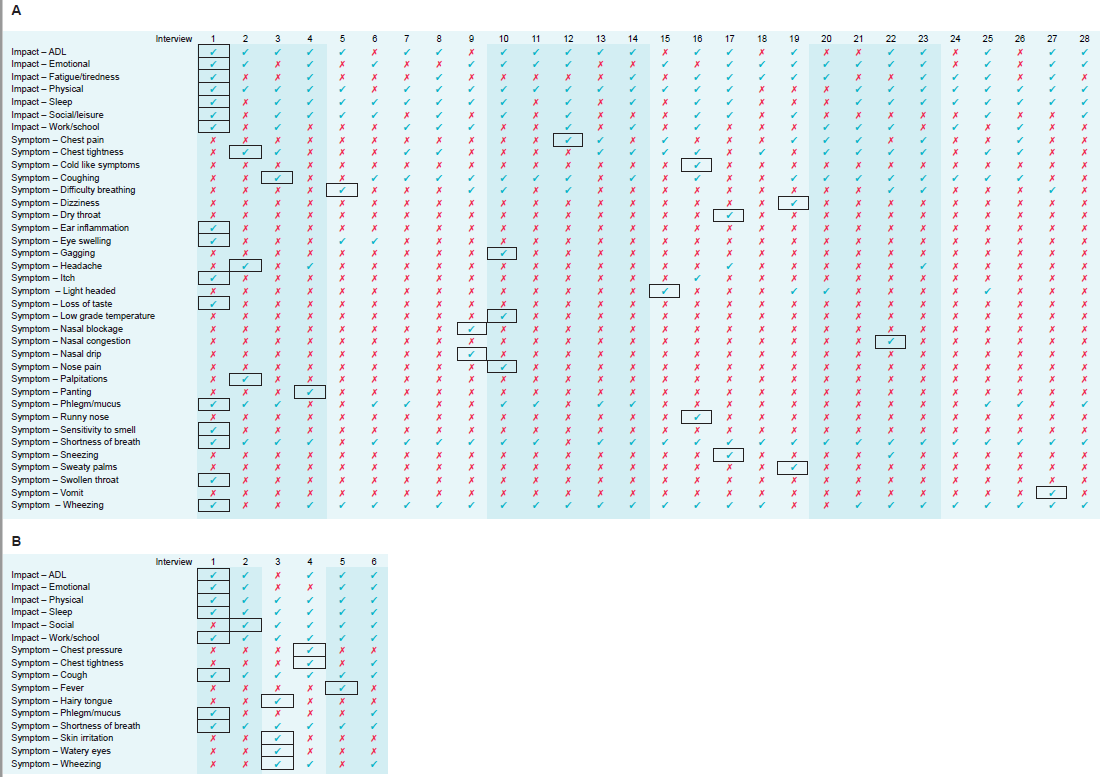
**

Note: All the presented concepts were reported spontaneously. **(A)** The majority (36/37 concepts, 97%) of the concepts were spontaneously elicited before the last set of five interviews. **(B)** 15/16 concepts (94%) of the concepts were spontaneously elicited before the last set of two interviews.

ADL, activities of daily living.
