# Supplementary material for: Patient experience of moderate asthma attacks: qualitative research in the USA and Germany
Source: J Patient Rep Outcomes. 2022 Nov 22;6:117. doi: 10.1186/s41687-022-00506-2 (PMC9681943; doi:10.1186/s41687-022-00506-2)
Supplement: Supplementary file 2 — Additional file 2. Table S1. [file 41687_2022_506_MOESM2_ESM.docx]

**Supplementary Tables**

**Supplementary Table 1: Target and actual recruitment quotas for the total population**

| **Patient characteristics** | | **Target (n)** | **Actual (n)** |
| --- | --- | --- | --- |
| **Age, years** | 18–30 | ≥5 | 8 |
|  | 31–55 | ≥5 | 15 |
|  | >55 | ≥5 | 5 |
| **Sex** | Male | ≥10 | 12 |
|  | Female | ≥10 | 16 |
| **Country** | USA | ≤24 | 20 |
|  | Germany | ≥8 | 8 |
| **Race** | White/Caucasian^a^ | ≥8 | 20 |
|  | Non-White/Non-Caucasian^a^ | ≥8 | 8 |
| **Education** | Completed high school or below only (i.e. not further college education or above) | ≥12 | 13 |
|  | Some college or above | ≥12 | 15 |
| **Asthma Control Test score** | Well controlled (≥20) | ≥4 | 5 |
|  | Not well-controlled (16–19) | ≥8 | 9 |
|  | Very poorly controlled (≤15) | ≥8 | 14 |
| **Medication step needed to maintain control** | GINA Step 2 | ≥5 | 8 |
|  | GINA Step 3 | ≥5 | 5 |
|  | GINA Step 4 | ≥5 | 14 |
|  | GINA Step 5 | N/A | 1 |

GINA, Global Initiative for Asthma.

^a^The authors want to acknowledge that the target to recruit ‘White/Caucasian and ‘Non-White/Non-Caucasian’ participants is reductive. We endeavour to conduct and report on research in a way that is inclusive and this involves highlighting ways we have failed to do so.
